# The Antitumor Constituents from *Hedyotis Diffusa* Willd

**DOI:** 10.3390/molecules22122101

**Published:** 2017-11-30

**Authors:** Changfu Wang, Xuegang Zhou, Youzhi Wang, Donghua Wei, Chengjie Deng, Xiaoyun Xu, Ping Xin, Shiqin Sun

**Affiliations:** 1 College of TCM, Guangdong Pharmaceutical University, No. 280 Outside Loop East Road of Higher Education Mega Center, Panyu District, Guangzhou 510006, China; wangchangfu831124@163.com; 2 College of Pharmacy, Harbin Medical University-Daqing, Daqing 163319, China; zhouxuegang1983@163.com (X.Z.); weidonghua66@sina.com (D.W.); fion8885@163.com (C.D.); 13136996624@sohu.com (X.X.); 3 Department of Medicinal Chemistry, School of Pharmacy, China Pharmaceutical University, Nanjing 210009, China; wangyouzhi@163.com

**Keywords:** *Hedyotis diffusa* Willd., antitumor, iridoid glycosides, cerebrosides

## Abstract

As a TCM, *Hedyotis diffusa* Willd. has been using to treat malignant tumors, and many studies also showed that the extracts from *Hedyotis diffusa* Willd. possessed evident antitumor activities. Therefore, we carried out chemical study on *Hedyotis diffusa* Willd. and investigated the cytotoxicity of the obtained compounds on a panel of eight tumor cell lines. As a result, four new compounds were isolated from *Hedyotis diffusa* Willd., including three iridoid glycosides of Shecaoiridoidside A–C (**1**–**3**) and a cerebroside of shecaocerenoside A (**4**). Also, six known iridoid compounds (**5**–**10**) were also obtained. The cytotoxicity of all compounds against human tumor cell lines of HL-60, HeLa, HCT15, A459, HepG2, PC-3, CNE-2, and BCG-823 were also evaluated in vitro. New compound **3** exhibited evident cytotoxicity to all tumor cell lines except the Hela, and the IC_50_ values are from 9.6 µM to 62.2 µM, while new compound **4** showed moderate cytotoxicity to all the cell lines, and the IC_50_ values are from 33.6 µM to 89.3 µM. By contrast, new compound **1** and known compound **9** showed moderate cytotoxicity to HCT15, A459, and HepG2 selectively. Known compound **7** also exhibited moderate cytotoxicity to HCT15 and A459 selectively.

## 1. Introduction

As an annual herb, Genus of *Hedyotis* has been widely distributed in subtropical area of the world [1]. 62 species of *Hedyotis* are distributed in China, among which more than 20 species are used as medicines, ingcluding *Hedyotis diffusa* Willd. (*H. diffusa* Willd.). *H. diffusa* Willd. has been recorded in Chinese pharmacopoeia (2015 edt) and possesses the efficacies of diuresis to reduce edema, clearing away the heat evil and detoxifying, and promoting blood circulation to arrest pain [2]. Clinically, *H. diffusa* Willd. has been using to treat urinary tract infection, tonsillitis, appendicitis, pharyngitis, hepatitis, dysentery, diarrhea, and snake bites [1,2,3]. But more importantly, *H. diffusa* Willd. also showed significant effective on malignant tumors of breast, gastric, colon, rectal, and ovarian [2,4,5]. The components of iridoids, triterpenes, flavonoids, lignans, anthraquinones, alkaloids, cerebrosides, coumarins, and sterols were discovered during the chemical studies of *H. diffusa* Willd. [2,6,7,8]. There are some differences of the chemical constituents if the *H. diffusa* Willd. grown in different parts of China. The contents of anthraquinones and iridoids in *H. diffusa* Willd. from Guangdong province were higher than the *H. diffusa* Willd. from the provinces of zhejiang, Jiangxi, Hubei, and Fujian. These include 2,7-dihydroxy-3-methyl anthraquinone, 2-hydroxy-3-methyl-1-methoxy anthraquinone, 2-hydroxy-3-methoxy-7-methoxy anthraquinone, 2-methyl-3-hydroxy anthraquinone, 2-methyl-3-hydroxy-4-methoxy anthraquinone, deacetyl asperulosidic acid, scandoside, *E*-6-*O*-*p*-coumaroyl scandoside methyl ester [9]. Various hepatoprotective, immunoloregulation, anti-tumor, anti-inflammatory, antibacterial, analgesia, sedative, and anti-oxidant activities can be found in pharmacological studies of *H. diffusa* Willd [3,6,10,11,12,13], but more studies found that the extracts from *H. diffusa* Willd. possessed evident anticancer activities [1,2,14,15,16,17,18,19,20]. *H. diffusa* Willd. has also been used to treat cancers adjuvantly for a long time in China. With increasing incidence and mortality in China, cancer has become the leading cause of death and caused serious public health problems. According to the latest report, in 2015, about 4,292,000 new cancer cases and 2,814,000 cancer deaths occurred in China, with lung cancer being the most common incident cancer and the leading cause of cancer death. Stomach, esophageal, and liver cancers were also commonly diagnosed and identified as leading causes of cancer death [21]. Therefore, screened active components from *H. diffusa* Willd. might be helpful. In this study, we carried out chemical study on *H. diffusa* Willd., and four new (**1**–**4**) along with six known (**5**–**10**) compounds were obtained. The structures of known compounds were determined by detailed NMR and ESI-MS spectra analyses, as well as comparing the data with the literature. In this paper, we describe the isolation of compounds **1**–**4** and elucidate their structures. The cytotoxic activity of all compounds against tumor cell lines of HL-60 (human leukemia cells), HeLa (human cervical cancer cells), HCT15 (human colon cancer cells), A459 (human lung cancer cells), HepG2 (human hepatoma cells), PC-3 (human prostate cancer cells), CNE-2 (human nasopharyngeal cancer cells), and BCG-823 (human gastric gland carcinoma cells) were also investigated in vitro. As a result, some active compounds will be screened, and the therapeutic basis of *H. diffusa* Willd. on tumors will also be revealed.

## 2. Results and Discussion

**Figure 1:**
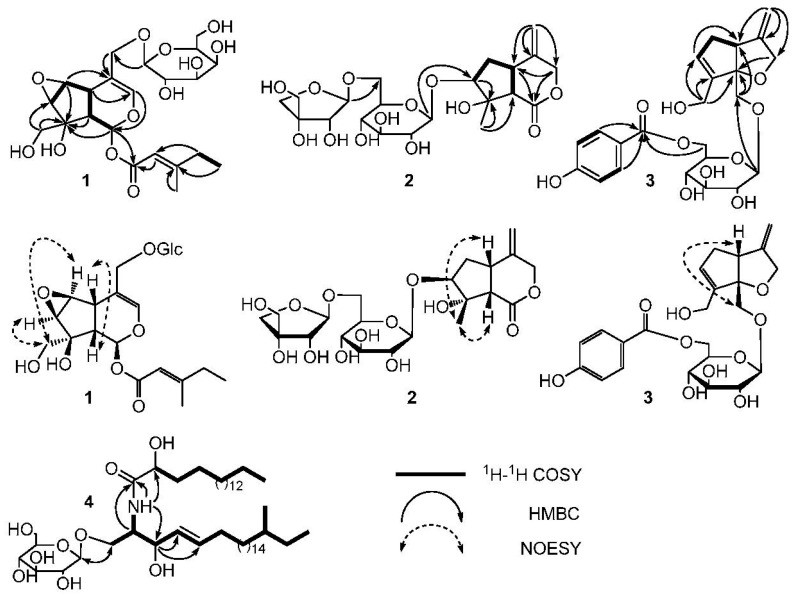
The ^1^H−^1^H Correlation Spectroscopy (COSY), key Heteronuclear Multiple Bond Correlation (HMBC) correlations of **1**–**4**, and Nuclear Overhauser Effect (NOE) correlations of **1**–**3**.

Compound **1** was obtained as a white amorphous powder. HRESIMS ([M + Na]^+^ *m*/*z* 511.1788, calc. for 511.1791) established the molecular formula of **1** as C_22_H_32_O_12_. Hydrolysis experiment of **1** liberated d-galactose which determined by GC-MS analysis. In the ^1^H-NMR spectrum of **1** (Table 1), signals of two methyl groups at *δ*_H_ 0.89 (3H, t, *J* = 7.4 Hz, H-5′) and 2.15 (3H, s, H-6′) could be observed. The *β*-configuration of galactopyranosyl moiety was confirmed by the coupling constant of H-1” (*J* = 8.1 Hz). The ^13^C-NMR and DEPT spectra of **1** (Table 2) showed 22 carbon signals, including six carbon signals for a *β*-d-galactopyranosyl moiety at *δ*_C_ 100.4, 72.6, 73.2, 69.2, 75.6, and 63.4, and a 4-methylsenecioyloxy group at *δ*_C_ 165.8, 114.6, 162.1, 33.8, 11.7, and 19.0. The left carbon signals were further identified by the 2D-NMR spectra of **1**. The HSQC and ^1^H−^1^H COSY spectra of **1** showed the coupling sequences of C(1)−C(9)−C(5)−C(6)−C(7) (Figure 1). The iridoids structure for **1** was established by the HMBC spectrum (Figure 1). The HMBC correlations from H-1” to C-11 and H-1 to C-1′ suggested that the galactopyranosyl moiety was located at C-11 and the 4-methylsenecioyloxy group was located at C-1.

**Figure 2:**
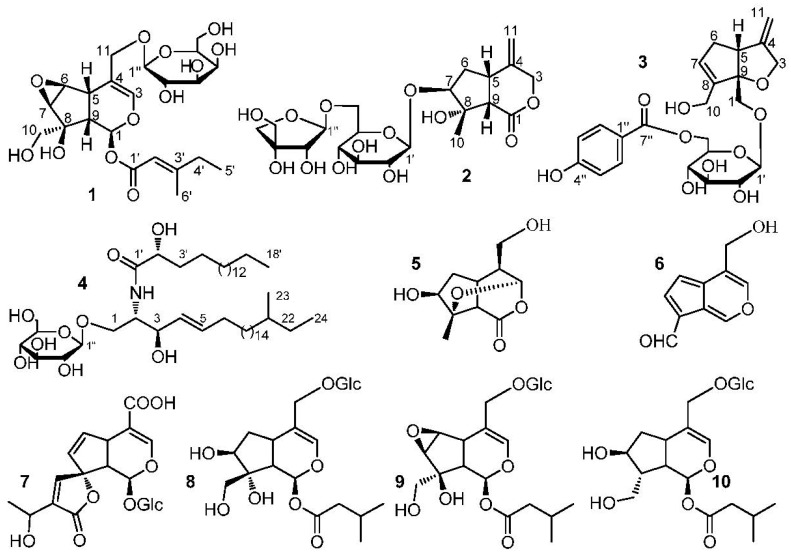
The structures of compounds **1**–**10**.

The stereo-configuration of **1** was determined by NOESY spectrum. The NOE correlations of H-5/H-9, H-7/H-10 and H-6/H-10, but the absence of correlations of H-5/H-1, H-5/H-7, H-5/H-10 and H-7/H-9 suggested that 8-OH, H-5 and H-9 were *β*-oriented, while H-1, H-6, H-7 and 8-CH_2_OH were *α*-oriented. Thus, the structure of **1** was established as (1*S*,5*S*,9*S*,6*S*,7*R*,8*S*)-8-hydroxy-8-hydroxymethyl-6,7-epoxylcyclopenta[*c*]pyran-1-*O*-4-methylsenecioyloxyl-11-hydroxymethyl-3-en 11-*O*-*β*-d-galactopyranoside and named as Shecaoiridoidside A (Figure 2).

Compound **2** was isolated as a white amorphous powder. HRESIMS ([M + Na]^+^ *m*/*z* 515.1737, calcd. 515.1741) determined the molecular formula of **2** as C_21_H_32_O_13_. Hydrolysis experiment of **2** liberated d-glucose and d-apiose which determined by GC-MS analysis. In the ^1^H-NMR spectrum of **2** (Table 1), signals of three methenyl groups at *δ* 3.34 (1H, m, H-5), 3.87 (1H, m, H-7), 3.08 (1H, d, *J* = 10.7 Hz, H-9), three methylene groups at *δ* 5.10 (1H, d, *J* = 11.0 Hz, H-3a), 4.44 (1H, d, *J* = 11.6 Hz, H-3b), 2.34 (1H, dd, *J* = 8.4, 13.4 Hz, H-6a), 2.19 (1H, m, H-6b), 5.08 (s, H-11a), 5.011 (s, H-11b), and a methyl group at *δ* 1.59 (3H, s, H-10) could be observed. The *β*-configuration of glucopyranosyl moiety was confirmed by the coupling constant of H-1′ (*J* = 7.8 Hz). The ^13^C-NMR and DEPT spectra of **2** (Table 2) showed 21 carbon signals, except for the 6 carbon signals at *δ*_C_ 99.9, 75.5, 78.7, 72.3, 78.3, 68.3 belong to a C-6′ substituted *β*-d-glucopyranosyl moiety and 5 carbon signals at *δ*_C_ 111.5, 76.2, 80.8, 75.4, 65.8 belong to a terminal *β*-d-apiofuranosyl moiety [22]. The left 10 carbon signals were similar to those of jatamanin A [23]. The main difference lies in the chemical shift value of C-7 in **2** was shifted downfield by 8.3 compared to that of jatamanin A, which confirmed that the *β*-d-glucopyranosyl moiety was located at C-7. The HSQC and ^1^H−^1^H COSY spectra of **2** showed the coupling sequence of C(9)−C(5)−C(6)−C(7) (Figure 1). The cyclopenta[*c*]pyran-type iridoid structure for **2** was established by the HMBC correlations from H-7 to C-8, H-7 to C-9, H-5 to C-1, and H-3 to C-5. The HMBC correlations from H-1” to C-6′ and H-1′ to C-8 suggested that the apiofuranosyl moiety was located at C-6′ and glucopyranosyl moieties was located at C-7 (Figure 1).

The stereo-configuration of **2** was determined by NOESY spectrum. The NOE correlations (Figure 2) of H-9/CH_3_-10 and H-5/CH_3_-10, but absence of the correlations of H-9/H-7 and H-5/H-7, suggested that H-5, H-9 and CH_3_-10 were *β*-oriented, while 8-OH and H-7 was α-oriented. Therefore, the structure of **2** was founded to be (5*S*,7*S*,8*S*,9*S*)-8-hydroxy-8-methyl-4-methylenehexahydrocyclo-penta[*c*]pyran-1(3*H*)-one 7-*O*-(6-*O*-*β*-d-apiofuranosyl)-*β*-d-glucopyranoside and named Shecaoiridoidside B (Figure 2).

Compound **3** was obtained as a white amorphous powder. HRESIMS ([M + Na]^+^ *m*/*z* 487.1576, calcd. 487.1580) established the molecular formula of **3** as C_23_H_2__8_O_10_. Hydrolysis experiment of **3** liberated d-glucose which determined by GC-MS analysis. In the ^1^H-NMR of **3** (Table 1), signals of two oxygenated methylenes at *δ* 3.96 (1H, d, *J* = 10.4 Hz, H-1a), 3.76(1H, d, *J* = 10.4 Hz, H-1b), 4.37 (1H, d, *J* = 12.6 Hz, H-3a) and 4.18 (1H, d, *J* = 12.6 Hz, H-3b), a nonoxygenated methylene at *δ* 2.74 (1H, m, H-6a) and 2.26 (1H, brd, *J* = 16.4 Hz, H-6b), a nonoxygenatedmethine at *δ* 3.25 (1H, m, H-5), and three olefinic protons at *δ* 5.78 (1H, brs, H-7), 4.91 (1H, d, *J* = 2.0 Hz, H-11a) and 4.92 (1H, d, *J* = 2.0 Hz, H-11b), and a p-substituted benzene protons at 7.88 (2H, d, *J* = 8.8 Hz) and 6.81 (2H, d, *J* = 8.8 Hz) were observed. The *β*-configuration of glucopyranosyl moiety was confirmed by coupling constant of H-1′ (*J* = 7.8 Hz). The ^13^C-NMR and DEPT spectra of **3** (Table 2) showed 23 carbon signals, except for the 6 carbon signals at *δ*_C_ 103.9, 74.8, 77.7, 72.3, 76.0, 65.2 belong to a C-6′ substituted *β*-d-glucopyranosyl moiety and 7 carbon signals at *δ*_C_ 122.4, 132.9 × 2, 116.6 × 2, 164.2, 167.8 belong to a *p*-hydroxybenzoyl moiety, the left 10 carbon signals were similar with those of patriridoside G [24]. The main difference lies in the signal at *δ*_C_ 12.1 (CH_3_-10) in patriridoside G was substituted by the signal at *δ*_C_ 59.3 (CH_2_-10) in **3**, which indicated that CH_3_-10 of patriridoside G was substituted by a hydroxyl group.

The HSQC and ^1^H−^1^H COSY spectra of **3** showed the coupling sequences of C-3/C-4/C-11, C-11/C-4/C-5/C-6/C-7/C-8/C-10, C-2”/C-3”, and C-5”/C-6” (Figure 2). HMBC correlations of from H-10 to C-7, C-8, and C-9, H-1 to C-5 and C-9, H-11 to C-3, C-4, and C-5, H-3 to C-9, H-1′ to C-1, H-6′ to C-7” and H-2”/H-6” to C-7” established the structure of **3** (Figure 1). The NOE correlation of H-5*β*/H-1 in NOESY spectra suggested a *β*-orientation for C-1 (Figure 1). As a result, the structure of **3** was identified as (5*R*,9*S*)-6-*O*-(6-*O*-4-hydroxybenzoyl-*β*-d-glucopyranosyl)-8-hydroxymethyl-4-methylene-4,5,6,9-tetrahydro-3*H*-cyclopenta[*b*]furan-9-yl-methanol and named Shecaoiridoidside C (Figure 2).

Compound **4** was obtained as white amorphous powder. HRESIMS *m*/*z* 828.6924 [M + H]^+^ (calc. for 828.6929) determined the molecular formula of **4** as C_48_H_93_NO_9_. Methanolysis experiment of **4** liberated d-glucose which determined by GC-MS analysis. In ^1^H- and ^13^C-NMR spectra of **4**, signals of anomeric proton *δ*_H_ (4.90, 1H, d, *J* = 7.6 Hz) and *δ*_C_ (105.6, 75.2, 78.6, 71.5, 78.7, and 62.6) indicated the presence of a *β*-d-glucopyranosyl moiety. The characteristics of a cerebroside with a 2-hydroxy fatty acid fraction in **4** could be confirmed by analyzing its ^1^H- and ^13^C-NMR data (Table 1 and Table 2). A fatty acid methyl ester (FAME) and a long-chain base (LCB) were obtained respectively by methanolysis of **4**. GC-MS analysis determined the structure of FAM as 2-hydroxyoctadecanoic acid methyl ester. The absolute configuration of C-2′*R* was determined by the specific rotation ${\left[\alpha \right]}_{D}^{22}$ = −4.8° (*c* 0.03, CHCl_3_) of the FAM [25]. The NMR data of C-2 and C-3 were compared with those of in literatures [26,27] and determined their stereo-configurations as 2*S* and 3*R*, respectively. The correlations of *δ*_H_ 4.77 (1H, m, H-3) with 131.6 (C-4) and 132.7 (C-5) in HMBC spectrum of **4** confirmed the olefinic bond was located in the LCB (Figure 2). The signals at *δ*_C_ 11.8 and 19.6 in ^13^C-NMR spectrum of **4** indicated the presence of a branched methyl group in **3**. To determine the position of the branched methyl group, the 1D-TOCSY spectrum was used and correlations of *δ*_H_ 4.22 (1H, m, H-1) with 5.86 (1H, m, H-4), 0.88 (3H, d, *J* = 6.4 Hz, CH_3_-23), and 0.86 (3H, t, *J* = 6.4 Hz, CH_3_-24) could be observed. Therefore, the branched methyl group was located in the LCB. The ^1^H- and ^13^C-NMR data (Table 1 and Table 2) were further assigned by the spectra of DEPT, HSQC, ^1^H-^1^H COSY, and HMBC. Thus, **4** was established as 1-*O*-*β*-d-glucopyranosyl-(2*S*,3*R*,4*E*)-2-[(2′*R*)-2-hydroxyloctadecanamideamino]-21-methyl-4-tetracosene-1,3-diol which was named as shecaocerenoside A (Figure 1). 

The known compounds were identified as jatamanin E (**5**) [28], 11-methoxyviburtinal (**6**) [29], 15-Demethylisoplumieride (**7**) [28], suspensolide F (**8**) [30], kanokoside A (**9**) [31], and patrinoside (**10**) [32] by comparing their physico-chemical constants and NMR spectroscopic data with those of in literatures (Figure 2).

The cytotoxicity of compounds **1**–**10** against tumor human cell lines of HL-60, HeLa, HCT15, A459, HepG2, PC-3, CNE-2, and BCG-823 were investigated in vitro. The MTT method was used to determine the IC_50_ values. New compound **3** exhibited evident cytotoxicity to all tumor cell lines except the Hela, and the IC_50_ values are from 9.6 μM to 62.2 μM, while new compound **4** showed moderate cytotoxicity to all the cell lines and the IC_50_ values are from 33.6 μM to 89.3 μM. By contrast, new compound **1** and known compound **9** showed moderate cytotoxicity to HCT15, A459, and HepG2 selectively. Known compound **7** also exhibited moderate cytotoxicity to HCT15 and A459 selectively (Table 3). Compounds **1** and **9** with the structural stem-nucleus 8-hydroxy-8-hydroxymethyl-6,7-epoxylcyclopenta[*c*]pyran-1-*O*-4-methylsenecioyloxyl-11-hydroxymethyl-3-en 11-*O*-*β*-d-glycoside were tend to show cytotoxicity to HepG2, which was consist with the reference reported [33]. While HCT15 was tend to sensitive to compound **3**. The cytotoxicity of sfingolipids has been reported in many references, and depend on its LCB, FAM, double bonds and glycosyl group to show moderate or weak activity to most of tumor cell lines [34,35,36,37], as well as compound **4**.

## 3. Materials and Methods

### 3.1. General

Column chromatographies such as Macroporous resin (AB-8 Crosslinked Polystyrene, Shanxi Lanshen Resin, Xi’an, China), silica gel (200–300 mesh, Hejie Technology Co. Ltd., Shanghai, China), and ODS-A (120A, 50 mm; DAISO, Kyoto, Japan) were used for isolations. Compounds were prepared on a preparative HPLC (Waters, Milford, MA, USA). Bruker AVANCE 400 MHz NMR instrument (Bruker SpectroSpin, Karlsruhe, Germany) was used to measure all the NMR spectra, including 1D-NMR and 2D-NMR spectra. Measured and analyzed the HRESIMS data was conducted on a Xero Q Tof MS spectrometer (Waters, Milford, MA, USA). IR Spectra data was recorded on FTIR-8400S (Shimadzu, Kyoto, Japan). The GC-MS (Angilent, Palo Alto, CA, USA) instrument was used to analysis the volatile derivatives from compounds. The growth of the tumor cell lines was monitored with a microplate reader (BMG FLUOStar OPTIMA, Ortenberg, Germany).

### 3.2. Plant Materials

The aerial part of *H. diffusa* Willd. was collected from Guangdong province of China and identified by Shuyuan Li of Guangdong Pharmaceutical University. The voucher specimen (No. 20160987) is deposited at the Herbarium of Guangdong Pharmaceutical University, Guangzhou, China.

### 3.3. Extraction and Isolation

The dried *H. diffusa* Willd. (10.0 Kg) were extracted two times (each for 2 h) with 75% EtOH (100 L) under reflux. The extract (1611 g) was suspended in water (15 L), and then extracted with petroleum ether (60–90 °C), EtOAc and *n*-butanol, respectively. Solvents were removed under vacuum to give extracts of petroleum ether (74.3 g), EtOAc (135.3 g), *n*-butanol (196.5 g), and remained water (1152.4 g). The EtOAc fraction (150.0 g) was subject to silica gel column column and eluted with a gradient of CH_2_Cl_2_/MeOH (30:1 to 0:1) to yield fractions of F_1_–F_6_. F_2_ (28.4 g) was further chromatographed on silica gel column and eluted with petroleum ether/EtOAc (15:1 to 1:1) to yield subfractions of A_1_–A_4_. The sub-fraction A_2_ (6.2 g) was repeated chromatographed on silica gel column and eluted with petroleum ether/EtOAc (5:1) to yield compound **4** (58 mg). F_3_ (30.6 g) was chromatographed on silica gel column and eluted with a gradient of CH_2_Cl_2_/MeOH (20:1 to 5:1) to yield sub-fractions B_1_–B_5_. B_2_ (10.4 g) was repeated chromatographed on silica gel column and eluted with CH_2_Cl_2_/MeOH (15:1) to yield compound **6** (46 mg). B_4_ (5.6 g) was repeated chromatographed on silica gel column and eluted with CH_2_Cl_2_/MeOH (8:1) to yield compound **5** (41 mg). F_4_ (62.4 g) was chromatographed on silica gel column and eluted with a gradient of CH_2_Cl_2_/MeOH (15:1–1:1) to yield sub-fractions C_1_–C_6_. C_3_ (11.2 g) was chromatographed on silica gel column and eluted with a gradient of CH_2_Cl_2_/MeOH (10:1 to 3:1), and then purified on a preparative HPLC with Hypersil-ODS II column (10 μm, 20 × 300 mm) eluted with MeOH/H_2_O (18%, flow rate 8 mL/min) to yield compounds **8** (48 mg, *t*_R_ = 15 min), **10** (57 mg, *t*_R_ = 27 min), **1** (62 mg, *t*_R_ = 31 min), and **9** (53 mg, *t*_R_ = 35 min). C_5_ (14.4 g) was chromatographed on silica gel column and eluted with CH_2_Cl_2_/MeOH (5:1), and then purified on a preparative HPLC with Hypersil-ODS II column (10 μm, 20 × 300 mm) eluted with MeOH/H_2_O (8%, flow rate 8 mL/min) to yield compounds **2** (48 mg, *t*_R_ = 11 min), **7** (43 mg, *t*_R_ = 18 min), and **3** (55 mg, *t*_R_ = 23 min).

*Shecaoiridoidside A* (**1**). white amorphous powder; ${\left[\alpha \right]}_{D}^{22}$ = −25.4 (*c* = 0.20, CH_3_OH); IR (KBr) *ν*_max_ 3433, 3384, 2921, 2871, 1723, 1648, 1455, 1353, 1252, 1082, 880 cm^−1^; ESIMS *m*/*z* 511 (100) [M + Na]^+^; HRESIMS [M + Na]^+^ *m*/*z* 511.1788 calc. 511.1791 for C_22_H_32_O_12_Na; ^1^H- and ^13^C-NMR data, see Table 1 and Table 2.

*Shecaoiridoidside B* (**2**). white amorphous powder, ${\left[\alpha \right]}_{D}^{22}$ + 109.4° (*c* 0.10, MeOH); IR (KBr) *ν*_max_ 3462, 3430, 2974, 2858, 1712, 1648, 1428, 1373, 1235, 1104 cm^−1^; ESIMS *m*/*z* 515 (100) [M + Na]^+^; HRESIMS [M + Na]^+^ *m*/*z* 515.1737, calcd. 515.1741 for C_21_H_32_O_13_Na; ^1^H- and ^13^C-NMR data, see Table 1 and Table 2.

*Shecaoiridoidside C* (**3**). white amorphous powder, ${\left[\alpha \right]}_{D}^{22}$ + 44.6° (*c* 0.12, MeOH); IR (KBr) *ν*_max_ 3518, 3421, 2875, 1674, 1447, 1384, 1325, 1169, 1080, 891, 595 cm^−1^; ESIMS *m*/*z* 487 (100) [M + Na]^+^; HRESIMS [M + Na]^+^ *m*/*z* 487.1576, calcd. 487.1580 for C_23_H_28_O_10_Na; ^1^H- and ^13^C-NMR data, see Table 1 and Table 2.

*Shecaocerenoside A* (**4**). white amorphous powder; ${\left[\alpha \right]}_{D}^{22}$ = +6.4 (*c* = 0.15, C_5_H_5_N); IR (KBr) *ν*_max_ 3411, 2941, 2838, 1635, 1532, 1455, 1162, 724 cm^−1^; ESIMS *m*/*z* 828 (100) [M + H]^+^; HRESIMS [M + H]^+^ *m*/*z* 828.6924 calc. 828.6929 for C_48_H_93_NO_9_H; ^1^H- and ^13^C-NMR data, see Table 1 and Table 2.

### 3.4. Acid Hydrolysis of ***1**–**3***

Acid hydrolysis experiment was carried out as the method in reference [24]. Briefly, the sugar residues were obtained by hydrolyzing of compounds **1**–**3** (2.0 mg) with 2 mol/L H_2_SO_4_ (2.0 mL), and then treated with trimethylchlorosilane, respectively. The sugar derivatives were further analyzed by GC-MS. As a result, the sugar derivatives from compounds **1** and **3** were determined to be d-galactose (*t*_R_ = 19.46 min) and d-glucose (*t*_R_ = 11.33 min), respectively. The sugar derivatives from compound **2** was determined to be d-glucose (*t*_R_ = 11.33 min) and d-apiose (*t*_R_ = 14.53 min).

### 3.5. Methanolysis of ***4***

Methanolysis of **4** was carried out according to the previous study [38]. In short, compound **4** (5.0 mg) was dissolved in in 82 % aqueous MeOH (20 mL) with 5% HCl and refluxed for 18 h. The FAME of **4** was obtained by extracting the reaction mixture with *n*-hexane. The FAME of **4** was a white amorphous powder, ${\left[\alpha \right]}_{D}^{22}$ = −4.8° (*c* 0.02, CHCl_3_). Analyzed the FAME by GC-MS and the characteristic fragment ions (*m*/*z* 314 [M]^+^, 256 [M − COOMe]^+^) were obtained. As a result, the FAME of **4** was identified as 2-hydroxyoctadecanoic acid methyl ester. The remained solution was analyzed by GC-MS and the monosaccharide of **4** was identified as d-glucose (*t*_R_ = 11.33 min). After that the remained solution was evaporated MeOH and the aqueous ammonia was added to adjust pH 9.0, and then extracted the solution with Et_2_O to obtain the LCB. The fragment ions of *m*/*z* 384 [M + H]^+^ and 366 [M − H_2_O + H]^+^ from ESIMS analysis led the LCB of **4** was identified as 2-aminotetracos-7-ene-1,3-diol (Figure 2).

### 3.6. Cytotoxicity Assay of Compounds ***1**–**10***

The cytotoxicity of all compounds against human tumor cell lines of HL-60, HeLa, HCT15, A459, HepG2, PC-3, CNE-2 and BCG-823 was assayed by 3-(4,5-dimethylthiazol-2-yl)-2,5-diphenyltetrazolium bromide (MTT) method in vitro. The assay protocol was conducted by previous published paper [24,39]. The tested compounds **1**–**10** were dissolved in DMSO and adjusted to the final concentrations from 1.0 μM to 300 μM by diluting with the growth medium. 5-Fluorouracil was used as the positive drug.

## 4. Conclusions

We investigated the chemical constituents of *H. diffusa* Willd. based on its clinical application of treating malignant tumors and 10 compounds were obtained, including three new iridoid glycosides and a new cerebroside. The structures of new compounds were identified as (1*S*,5*S*,9*S*,6*S*,7*R*,8*S*)-8-hydroxy-8-hydroxymethyl-6,7-epoxylcyclopenta[*c*]pyran-1-*O*-4-methylsenecioyl-oxyl-11-hydroxymethyl-3-en 11-*O*-*β*-d-glucopyranoside (**1**), (5*S*,7*S*,8*S*,9*S*)-8-hydroxy-8-methyl-4-methylenehexahydrocyclopenta[*c*]pyran-1(3*H*)-one 7-*O*-(6-*O*-*β*-d-apiofuranosyl)-*β*-d-glucopyrano-side (**2**), (5*R*,9*S*)-6-*O*-(6-*O*-4-hydroxybenzoyl-*β*-d-glucopyranosyl)-8-hydroxymethyl-4-methylene-4,5,6,9-tetrahydro-3*H*-cyclopenta[*b*]furan-9-yl-methanol (**3**), and 1-*O*-*β*-d-glucopyranosyl-(2*S*,3*R*,4*E*) -2-[(2′*R*)-2-hydroxyloctadecanamideamino]-21-methyl-4-tetracosene-1,3-diol (**4**), respectively. Antitumor assays in vitro discovered cytotoxic compounds **1**, **3**, **4**, **7**, and **9**, especially found that new compound **3** exhibited evident cytotoxicity to all tumor cell lines except the Hela.

## Figures and Tables

**Table 1:** ^1^H-NMR data of compounds **1**–**4** (400 MHz, δ in ppm, *J* in Hz).

1 ^a^		2 ^a^	3 ^a^	4 ^b^	
H	*δ*_H_ (*J*, Hz)	*δ*_H_ (*J*, Hz)	*δ*_H_ (*J*, Hz)	H	*δ*_H_ (*J*, Hz)
1	6.41, d (2.0)		3.96, d (10.4); 3.76, d (10.4)	NH	8.35, d, (8.4)
3	6.40, brs	5.10, d (11.0) 4.44, d (11.6)	4.37, d (12.6); 4.18, d (12.6)	1	4.22, m 4.72, m
5	3.09, brd (8.5)	3.34, m	3.25, m	2	4.78, m
6	4.04, d (2.5)	2.34, dd (8.4, 13.4); 2.19, m	2.74, m; 2.26, brd (16.4)	3	4.77, m
7	3.36, d (2.5)	3.87, m	5.78, brs	4	5.86, m
9	2.05, m	3.08, d (10.7)		5	5.98, m
10	3.69, d (2.8)	1.59, s	4.11, brd (10.0)	6	2.06, m
11	4.21, d (11.6); 4.35, d (11.6)	5.08, 5.11, s	4.92, 4.91, d (2.0)	7–22	1.16–1.42, brs
1′		4.41, d (7.8)	4.72, d (7.8)	23	0.88, d (6.4)
2′	5.62, s	3.16, t (8.2)	3.27, m	24	0.86, t (6.4)
3′		3.30, m	3.43, m	2′	4.61, m
4′	2.16, m	3.29, m	3.40, m	3′	1.85, m
5′	0.89, t (7.4)	3.34, m	3.62, m	4′	1.73, m; 1.16–1.42, brs
6′	2.15, s	3.59, 3.95, m	4.60, brd, (11.6); 4.42, dd, (11.8, 4.8)	5′–17′	1.16–1.42, brs
1””	4.72, d (8.1)	5.01, d (1.7)		18′	0.88, t (6.4)
2”	3.35, m	3.87, m	7.88, d (8.8)	1”	4.90, d, (7.6)
3”	4.05, m		6.81, d (8.8)	2”	4.02, m
4”	3.49, m	3.95, 3.75, m		3”	4.22, m
5”	3.59 (1H, m)	3.58, s	6.81, d (8.8)	4”	4.22, m
6”	3.67, m; 3.86, dd (1.5, 11.5)		7.88, d (8.8)	5”	3.88, m
				6”	4.36, 4.50, m

^a^ Measured in CD_3_OD at 30 °C; ^b^ Measured in C_5_D_5_N-*d*_5_ at 30 °C.

**Table 2:** ^13^C-NMR data of compounds **1**–**4** (100 MHz, *δ* in ppm).

1 ^a^		2 ^a^		3 ^a^		4 ^b^	
C	*δ*_C_	C	*δ*_C_	C	*δ*_C_	C	*δ*_C_
1	90.8, CH	1	175.2, C	1	72.8, CH_2_	1	70.2, CH_2_
3	142.4, CH	3	71.5, CH_2_	3	72.8, CH_2_	2	54.5, CH
4	109.8, C	4	144.5, C	4	156.2, C	3	72.3, CH
5	35.4, CH	5	41.2, CH	5	49.8, CH	4	131.6, CH
6	59.9, CH	6	40.0, CH_2_	6	39.2, CH_2_	5	132.7, CH
7	60.3, CH	7	90.1, CH	7	131.4, CH	6	34.2, CH_2_
8	80.2, C	8	86.1, C	8	144.4, C	7-20	29.5–30.5, CH_2_
9	43.6, CH	9	54.2, CH	9	99.8, C	21	35.7, CH
10	67.2, CH_2_	10	22.5, CH_3_	10	59.3, CH_2_	22	30.5, CH_2_
11	69.8, CH_2_	11	113.8, CH_2_	11	105.4, CH_2_	23	19.6, CH_3_
1′	165.8, C	1′	99.9, CH	1′	103.9, CH	24	11.8, CH_3_
2′	114.6, CH	2′	75.5, CH	2′	74.8, CH	1′	175.6, C
3′	162.1, C	3′	78.7, CH	3′	77.7, CH	2′	72.5, CH
4′	33.8, CH_2_	4′	72.3, CH	4′	72.3, CH	3′	35.8, CH_2_
5′	11.7, CH_3_	5′	78.3, CH	5′	76.0, CH	4′	26.2, CH_2_
6′	19.0, CH_3_	6′	68.3, CH_2_	6′	65.2, CH_2_	5′-15′	29.5–30.5, CH_2_
1”	100.4, CH	1”	111.5, CH	1”	122.4, C	16′	32.2, CH_2_
2”	72.6, CH	2”	76.2, CH	2”	132.9, CH	17′	22.8, CH_2_
3”	73.2, CH	3”	80.8, C	3”	116.6, CH	18′	14.2, CH_3_
4”	69.2, CH	4”	75.4, CH_2_	4”	164.2, C	1”	105.6, CH
5”	75.6, CH	5”	65.8, CH_2_	5”	116.6, CH	2”	75.2, CH
6”	63.4, CH_2_			6”	132.9, CH	3”	78.6, CH
				7”	167.8, C	4”	71.5, CH
						5”	78.7, CH
						6”	62.6, CH_2_

^a^ Measured in CD_3_OD at 30 °C; ^b^ Measured in C_5_D_5_N-*d*_5_ at 30 °C.

**Table 3:** In vitro antitumor activity of compounds **1**–**10** in a panel of 8 tumor cell lines.

Compounds	HL-60	Hela	HCT15	A459	HepG2	PC-3	CNE-2	BGC-823
**1**	>100.0	>100.0	87.6 ± 1.2	77.7 ± 1.6	37.6 ± 1.4	>100.0	>100.0	>100.0
**2**	>100.0	>100.0	>100.0	>100.0	>100.0	>100.0	>100.0	>100.0
**3**	17.1 ± 0.7	62.2 ± 0.5	9.6 ± 0.8	14.8 ± 0.9	11.4 ± 1.6	26.2 ± 1.3	21.5 ± 0.6	13.4 ± 1.1
**4**	74.8 ± 1.3	89.3 ± 1.8	37.3 ± 1.5	33.6 ± 1.1	49.5 ± 1.4	64.0 ± 0.9	55.2 ± 1.1	44.1 ± 1.7
**5**	>100.0	>100.0	>100.0	>100.0	>100.0	>100.0	>100.0	>100.0
**6**	>100.0	>100.0	>100.0	>100.0	>100.0	>100.0	>100.0	>100.0
**7**	>100.0	>100.0	71.3 ± 1.2	50.4 ± 1.1	>100.0	34.2 ± 1.3	>100.0	>100.0
**8**	>100.0	>100.0	89.8 ± 1.2	91.3 ± 0.7	>100.0	>100.0	>100.0	>100.0
**9**	>100.0	>100.0	96.1 ± 1.6	78.3 ± 0.8	97.9 ± 1.4	>100.0	>100.0	>100.0
**10**	>100.0	>100.0	>100.0	>100.0	>100.0	>100.0	>100.0	>100.0
5-Fluorouracil	7.5 ± 0.6	10.4 ± 0.4	4.7 ± 0.4	14.7 ± 1.1	22.8 ± 1.4	13.2 ± 0.7	11.6 ± 0.8	17.8 ± 0.7

Key: All results are expressed as IC_50_ values in μM. Compounds with IC_50_ > 100 μM were inactive for the tumor cell lines.
